# *Staphylococcus caprae* native mitral valve infective endocarditis

**DOI:** 10.1099/jmmcr.0.005065

**Published:** 2016-10-27

**Authors:** T'ng Choong Kwok, Jennifer Poyner, Ewan Olson, Peter Henriksen, Oliver Koch

**Affiliations:** ^1^​ Edinburgh Heart Centre, Western General Hospital, Edinburgh, Scotland; ^2^​ Clinical Microbiology, Royal Infirmary Edinburgh, Edinburgh, Scotland; ^3^​ Regional Infectious Diseases Unit, Western General Hospital, Edinburgh, Scotland

**Keywords:** *Staphylococcus caprae*, native valve, coagulase negative staphylococcus, occult prosthetic metalwork infection, conservative management

## Abstract

**Introduction:**

*Staphylococcus caprae* is a rare cause of infective endocarditis. Here, we report a case involving the native mitral valve in the absence of an implantable cardiac electronic device.

**Case presentation:**

A 76-year-old man presented with a 2 week history of confusion and pyrexia. His past medical history included an open reduction and internal fixation of a humeral fracture 17 years previously, which remained non-united despite further revision 4 years later. There was no history of immunocompromise or farm-animal contact. Two sets of blood culture bottles, more than 12 h apart, were positive for *S. caprae*. Trans-thoracic echocardiography revealed a 1×1.2 cm vegetation on the mitral valve, with moderate mitral regurgitation. Due to ongoing confusion, he had a magnetic resonance imaging brain scan, which showed a subacute small vessel infarct consistent with a thromboembolic source. A humeral SPECT-CT (single-photon emission computerized tomography-computerized tomography) scan showed no clear evidence of acute osteomyelitis. Surgical vegetectomy and mitral-valve repair were considered to reduce the risk of further systemic embolism and progressive valve infection. However, the potential risks of surgery to this patient led to a decision to pursue a cure with antibiotic therapy alone. He remained well 3 months after discharge, with repeat echocardiography demonstrating a reduction in the size of the vegetation (0.9 cm).

**Conclusion:**

Management of this infection was challenging due to its rarity and its unclear progression, complicated by the dilemma surrounding surgical intervention in a patient with a complex medical background.

## Introduction

Native valve endocarditis is rare in its own right, but much rarer when caused by coagulase-negative staphylococci (7.8 %) (Chu *et al.*, 2008) in the absence of an implantable cardiac electronic device (ICED) and history of intravenous (i.v.) drug use. *Staphylococcus caprae* is a coagulase-negative staphylococcus originally isolated in goats, but increasingly identified in nosocomial and community-acquired infections, especially human bone and joint infections (Shuttleworth *et al.*, 1997; Seng *et al.*, 2014). Here, we report a rare case of *S. caprae* native mitral valve infective endocarditis in the absence of an ICED.

## Case report

A 76-year-old man presented with a 2 week history of fever and dry cough. His symptoms were initially thought to be secondary to an upper respiratory tract infection and a viral throat swab had been PCR positive for Rhinovirus. However, when he started developing rigors and became confused to time and place, hospital admission was arranged by his general practitioner.

His past medical history consisted of hypertension with a previous echocardiogram a year prior showing good left ventricular systolic function, with mild left ventricular hypertrophy and mild mitral regurgitation. He previously underwent transurethral resection of the prostate for prostatism, and a total colectomy and ileostomy for Crohn’s disease. Furthermore, he had sustained a left humeral fracture 17 years previously requiring open reduction and internal fixation. Due to non-union, he underwent revision surgery 4 years later with replating and bone grafting of the non-union. This was complicated by an infection of the bone graft donor site requiring extended antibiotic therapy. Three months prior to his current presentation, he had developed pain in his left shoulder and humerus after lifting a heavy case. A computerized tomography (CT) scan of the left humerus at that point had shown persistent non-union of the previous fracture site.

## Investigations

**Fig. 1.**
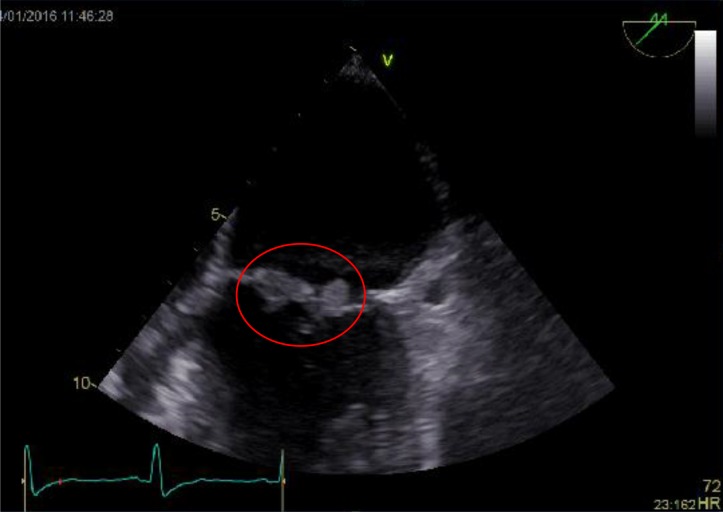
The mitral valve vegetation shown on a trans-thoracic echocardiograph (circled in red).

Admission blood tests revealed a white cell count of 10.4×10^9^ cells l^−1^ with an isolated neutrophilia of 8.47×10^9^ neutrophils l^−1^, and a C-reactive protein level of 180 mg l^−1^. Two sets of blood culture bottles, taken more than 12 h apart prior to commencing antibiotics, were positive for *S. caprae*, identified by matrix-assisted laser desorption/ionization time-of-flight mass spectrometry (MALDI-TOF) (with a Bruker MALDI-TOF mass spectrometer). Antibiotic sensitivity testing performed with a VITEK 2 system (bioMérieux) revealed that the organism was sensitive to flucloxacillin, gentamicin, rifampicin, vancomycin, teicoplanin and daptomycin, but resistant to penicillin. Further blood cultures taken after commencing i.v. flucloxacillin remained negative. The patient underwent a trans-thoracic echocardiogram revealing a 1×1.2 cm vegetation on the mitral valve (Fig. 1), with moderate mitral regurgitation.

**Fig. 2.**
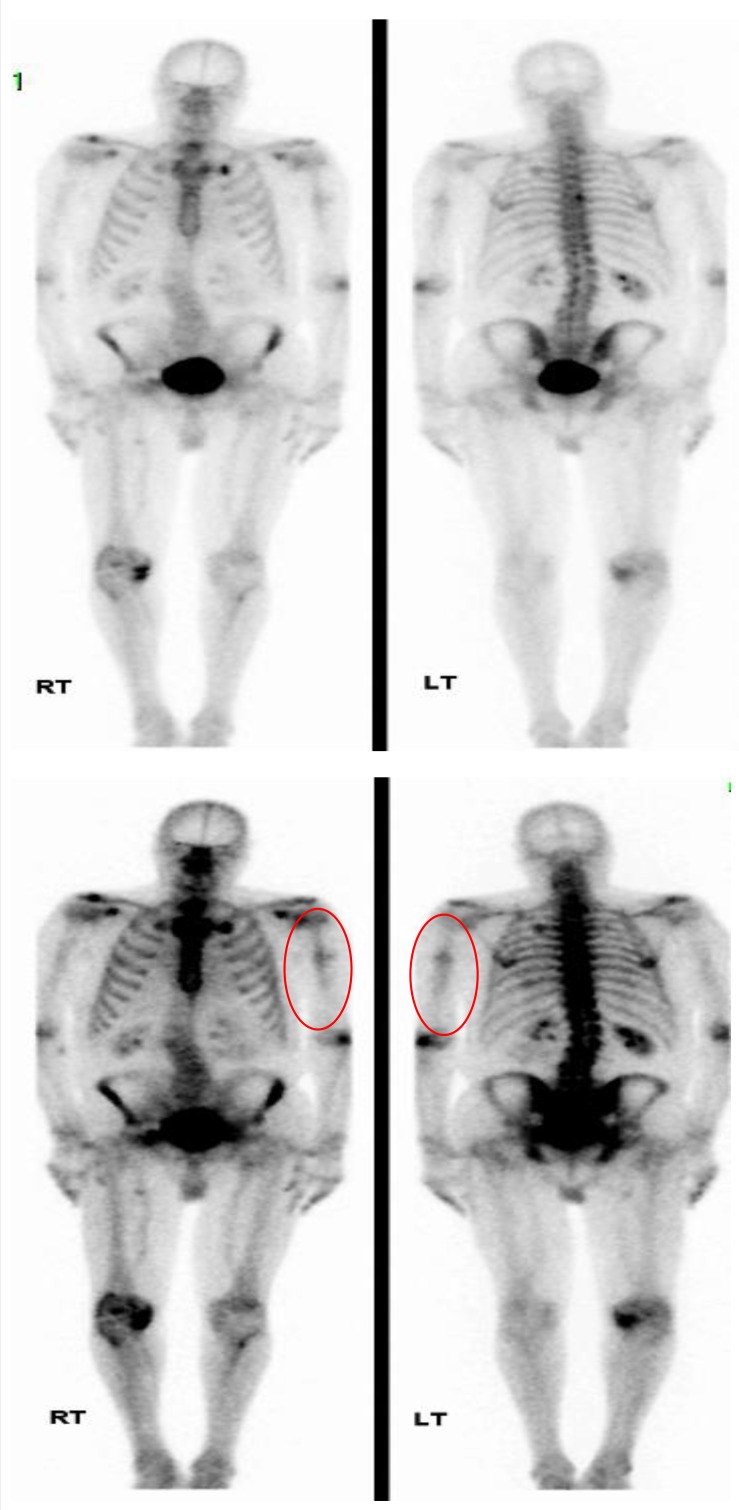
Bone scintigram revealing mild increased accumulation at the left humeral fracture site on late static bone, but no evidence of active osteomyelitis (circled in red). There is also benign degenerate uptake noted at various levels in the scoliotic spine and in the shoulder girdle, as well as the knee.

Due his confusion, a magnetic resonance imaging brain scanning was performed, which showed a subacute small vessel infarct of the left frontal lobe consistent with thromboembolism suggestive of septic emboli. A nuclear medicine whole body bone scan with thoracic SPECT (single-photon emission computerized tomography)-CT scan (Fig. 2) was undertaken to assess for any evidence of active osteomyelitis given the previously described association between this organism and bone and joint infections. No evidence of pathological uptake was identified.

## Diagnosis

*S. caprae* mitral valve endocarditis was diagnosed according to the Modified Duke’s criteria (Baddour *et al.*, 2015) on the basis of diagnostic trans-thoracic echocardiogram findings as the major criterion, and positive blood cultures, fever and vascular phenomena as minor criteria.

## Treatment

The patient was managed with 6 weeks of i.v. flucloxacillin (2g four times per day). In light of the size of the vegetation demonstrated on the echocardiogram, plus evidence of cerebral embolization, discussion with cardiothoracic surgeons was sought. The possibilities of surgical vegetectomy and mitral valve repair were considered to reduce the risk of further embolization and progression of infection. After 2 weeks of i.v. antibiotic the patient remained confused, limiting the possibility of informed consent for early surgical intervention. His clinical condition improved with cognition returning to baseline by 3 weeks. While the SPECT-CT scan had been normal, it was felt that this did not completely rule out the possibility of indolent deep-seated infection surrounding the previous humeral fracture site. Surgical intervention was again discussed, this time with the patient. The possibility or removing metalwork from his humerus was considered, but the risks of fracture site instability, non-union and subsequent loss of function in his arm were considered too great. His continued improvement on antibiotic therapy, together with uncertainty surrounding occult infection in his left humeral metalwork, led to a decision to pursue antibiotic therapy.

## Outcome and follow-up

He was managed conservatively with i.v. flucloxacillin, and a repeat trans-thoracic echocardiogram post-completion of i.v. antibiotics identified a reduction in vegetation size to 0.9×0.7 cm. He remained well and apyrexial at a 3 month clinic follow-up, with no further episodes of embolization.

## Discussion

*S. caprae* has only sporadically been reported in the literature. The natural history of this infection remains unclear. A case series (Seng *et al.*, 2014) documented 22 cases of *S. caprae* bone and joint infection, associated with orthopaedic foreign material. Of the 22, 15 cases (68 %) were identified as late infections occurring over a year after implantation of the material. This lends some support to the possibility of a deep-seated infection as a possible source of infective endocarditis.

The only evidence of *S. caprae* causing infective endocarditis found in literature was a case report published in 1995 (Vandenesch *et al.*, 1995). This involved a mitral valve with no ICED *in situ*, which was managed with early surgical removal of a large vegetation with the valve left *in situ*, followed by 2 weeks of i.v. vancomycin, resulting in excellent recovery. Other infrequent human *S. caprae* infections described in the literature include urinary tract infection, sepsis and acute otitis externa (Ross *et al.*, 2005).

Despite negative imaging, occult infection of the metalwork in the patient’s humerus could not be ruled out initially. There was uncertainty with respect to the benefits of surgical intervention, given that the patient’s clinical status and inflammatory markers had continued to improve with antibiotics alone. Furthermore, the British Society for Antimicrobial Chemotherapy guidelines (Gould *et al.*, 2012) suggest that aortic or mitral valve infective endocarditis with a large vegetation of more than 10 mm, resulting in one or more embolic episodes, warrants urgent surgery. A recent meta-analysis (Narayanan *et al.*, 2016) concluded that early surgical intervention (less than 3 weeks from the commencement of antibiotics) is associated with lower mortality [OR 0.41 (*P*<0.001)] in matched groups. There is, however, a non-significant trend to higher rates of re-infection with early surgery. While, on the whole, early surgical intervention is desirable in the management of infective endocarditis presenting with a large vegetation burden and embolic phenomena, clearly each patient’s individual circumstances need to be considered. Our patient made a full recovery with conservative therapy using antibiotics alone.
